# NG2 Glia and Cellular Crosstalk in Health and Disease: Focus on Spinal Cord Injury

**DOI:** 10.3390/pathophysiology33020038

**Published:** 2026-06-01

**Authors:** Ilyas Kabdesh, Aizilya Bilalova, Yana Mukhamedshina, Yuri Chelyshev

**Affiliations:** 1 OpenLab Gene and Cell Technologies, Institute of Fundamental Medicine and Biology, Kazan Federal University, 420021 Kazan, Russia; ayzrbilalova@kpfu.ru (A.B.); yomuhamedshina@kpfu.ru (Y.M.); 2 Division of Medical and Biological Sciences, Tatarstan Academy of Sciences, 420013 Kazan, Russia; 3 Department of Histology, Cytology and Embryology, Kazan State Medical University, 420008 Kazan, Russia; chelyshev-kzn@yandex.ru

**Keywords:** spinal cord injury, NG2 glia, NG2/CSPG4, OPCs, glial scar, cell–cell interactions

## Abstract

NG2 glia, also known as oligodendrocyte progenitor cells, represent a unique population of glial cells characterized by dynamic morphology and the ability to extend branched processes that actively contact neurons and other cellular elements. These structural and functional interactions enable NG2 glia to contribute to the regulation of axonal excitability, electrical activity, and axonal architecture. Unlike most other glial cells, NG2 glia receive direct synaptic input from neurons and can generate action potentials, defining their distinctive physiological status. A particularly important feature of this cell population is the expression of the chondroitin sulfate proteoglycan NG2/CSPG4, which serves as a key molecular marker and plays an essential role in intercellular interactions. Following spinal cord injury (SCI), NG2 glia rapidly become activated, undergo phenotypic changes, and engage in extensive interactions with neurons, astrocytes, microglia, and endothelial cells. These interactions form a complex regulatory network that influences both the severity of secondary injury and the effectiveness of remodeling and repair processes. Mechanisms of particular importance include the secretion of chondroitin sulfate proteoglycans and alterations in extracellular matrix properties. Finally, this review highlights potential therapeutic approaches aimed at modulating NG2 glial activity and their intercellular interactions. The focus is on strategies designed to reduce the inhibitory effects of proteoglycans while enhancing the remyelinating and neuroprotective potential of these cells, thereby opening new perspectives for regenerative therapies after SCI.

## 1. Introduction

Spinal cord injury (SCI) induces a profound functional deficit of neuronal networks, resulting in severe motor and sensory impairments. The adult central nervous system (CNS) possesses a low regenerative potential: neurogenesis is limited, and axonal repair is highly inefficient, which complicates reparative processes after injury. The primary reasons for this are the reduced intrinsic capacity for cellular regeneration and the formation of an inhibitory microenvironment in response to trauma [1,2]. For a long time, research on SCI primarily focused on the reactivity of astrocytes and microglia, whereas NG2 glia—also referred to as oligodendrocyte progenitor/precursor cells (OPCs), polydendrocytes, synantocytes, O2A or NG2 cells—did not receive comparable attention. However, over the past decade, NG2 glia have been recognized as the fourth type of glial cell in the CNS, and increasing attention has been drawn to their contribution to the inhibitory post-traumatic microenvironment, including their association with CSPG-rich scar components and their interactions with dystrophic axons in the injured spinal cord [3,4,5,6].

SCI triggers a cascade of pathological events, including rapid oligodendrocyte death, demyelination of surviving axons, and disruption of axonal conduction. In parallel, resident OPCs become activated, proliferate, and contribute to remyelination, although this process is often incomplete. At the same time, the formation of a glial scar and accumulation of extracellular matrix inhibitors create a microenvironment that limits axonal regeneration. These opposing processes (remyelination and scar-associated inhibition) place NG2 glia at a central position in the balance between repair and regeneration failure after SCI.

NG2 glia constitute approximately 5–10% of all cells in the adult central nervous system (CNS) [7]. These cells are highly heterogeneous, with differences in proliferation dynamics, electrophysiological properties, and fate potential depending on their anatomical location and developmental context [8]. NG2 glia are widely distributed throughout the CNS from the late stages of prenatal development and persist throughout life [8,9]. These NG2^+^ cells express chondroitin sulfate proteoglycan 4 (NG2/CSPG4) and platelet-derived growth factor receptor alpha (PDGFRα), which serve as the principal markers of this population under both physiological and pathological conditions. However, neither NG2/CSPG4 nor PDGFRα is strictly cell type-specific. Under physiological conditions, NG2/CSPG4 expression has been reported in several cell types, including neurons, mesenchymal stem cells, endothelial cells, osteoblasts, melanocytes, and smooth muscle cells. Its expression and function are influenced by multiple signaling pathways, including Notch, Hedgehog, and Wnt, although these regulatory mechanisms have been characterized in diverse cellular contexts and are not always directly demonstrated in CNS OPCs. In addition, NG2/CSPG4 has been implicated in processes such as cell migration, proliferation, and cell–cell interactions, including contacts between NG2 glia and neurons that may influence synaptic function [10]. NG2/CSPG4 exerts its functions through its extracellular domain, which interacts with more than 40 ligands, and through its cytoplasmic tail, which regulates signaling pathways linked to cytoskeletal organization. PDGFRα-mediated signaling has been reported to be altered in cells lacking NG2/CSPG4, suggesting a functional interaction between these two molecules, although this observation was not made in CNS OPCs [11]. The primary extracellular ligands of NG2/CSPG4 are components of the extracellular matrix (ECM). Early biochemical studies demonstrated that the ectodomain of NG2/CSPG4 can interact with ECM proteins, including tenascin-C, laminin 111, perlecan, and collagens [6,12]. More recent studies have further supported these interactions in different cellular contexts [13,14]. This relevance becomes particularly evident in the context of SCI, where many of the intrinsic properties of NG2 glia acquire new functional significance. Their rapid reactivity, sensitivity to extracellular signals, and capacity to remodel the ECM place them among the earliest responders to trauma-induced changes in the microenvironment [15,16]. In addition, NG2 glia dynamically adjust their proliferation and morphology in response to injury-related cues [15,17], while their secretory profile and interactions with extracellular matrix components contribute to tissue remodeling and scar formation [18]. These injury-induced transformations make NG2 glia active regulators of post-traumatic outcomes, underscoring the need to examine their functions specifically within the SCI context.

This review is focused on the role of NG2 glial cells in both physiological and pathological contexts, with a particular emphasis on SCI. In the first part, we examine the functional characteristics of NG2 glia under normal conditions. We then analyze their role and intercellular interactions in the context of SCI. Finally, we discuss current and emerging therapeutic approaches aimed at modulating the activity of these cells to enhance neuronal network recovery.

## 2. NG2 Glia and Their Function in Normal Environments

In the adult spinal cord, NG2 glia (OPCs) predominantly give rise to oligodendrocytes and constitute the dominant proliferating glial population under physiological conditions [19,20]. Their lineage potential in this region is considerably more restricted than in the developing CNS, and current lineage-tracing evidence indicates that adult spinal cord NG2 glia do not spontaneously generate neurons or astrocytes in vivo, but instead self-renew and generate mature oligodendrocytes [19]. Rather than behaving as broadly multipotent progenitors, they maintain a relatively stable progenitor state characterized by low-level proliferation and region-dependent responsiveness to local cues, including PDGF signaling [19,20]. Although broader differentiation capacities have been demonstrated in embryonic tissue, early postnatal stages, or in vitro, these findings do not reflect the physiological behavior of NG2 glia in the adult spinal cord. For example, BMP signaling has been shown to induce astrocytic differentiation of OPCs under in vitro conditions [21]. Under normal adult spinal cord conditions, their primary role is to sustain oligodendrogenesis and support white-matter homeostasis. Thus, these baseline properties of NG2 glia are essential for understanding their behavior in pathological conditions, including SCI, where these cells become rapidly activated and functionally reprogrammed.

In a recent study based on spatial transcriptomics and snRNA-seq, NG2 glia within the OPC pool were shown to segregate into subpopulations with distinct metabolic and myelinating activities in both human and mouse spinal cord [22]. Thus, it is important to emphasize that NG2 glia and OPCs are overlapping but not identical populations, and their phenotypic delineation requires an integrative approach combining transcriptomic, marker-based, and functional characteristics. In the present review, we use the term “NG2 glia” as a general designation for this cell population, whereas the term “OPCs” is employed in cases where it was explicitly used by the original authors or reflects a specific research context.

Several subtypes of NG2 glia have been described and classified according to different criteria. For instance, NG2 glia can be categorized as perivascular, parenchymal, or intermediate [23,24] and may also differ by their localization in gray or white matter, as well as by the expression levels of various receptors and ion channels [25]. Perivascular NG2 glia extend thin processes that ensheath microvessels, whereas parenchymal cells are located at some distance from them. In the adult brain, but not in the spinal cord, NG2 glia in gray matter are characterized by longer cell cycle duration, low proliferation rates, and slow differentiation. By contrast, in white matter, these glial cells proliferate more actively, exhibit shorter cell cycles, and differentiate at higher rates, giving rise to mature oligodendrocytes [26,27]. Such heterogeneity may become functionally relevant after SCI, where region-specific NG2 glial responses contribute differently to repair and inhibition.

Taken together, under physiological conditions, NG2 glia can be broadly considered according to their anatomical position, regional localization, and functional state. Parenchymal NG2 glia represent the classical OPC-like population involved in oligodendrocyte lineage maintenance and myelin homeostasis, whereas perivascular NG2 glia are closely associated with microvessels and may participate in vascular niche-related signaling. Intermediate forms may link parenchymal and vascular compartments, although their functional significance remains less clearly defined. In addition, white matter NG2 glia generally show higher proliferative and oligodendroglial differentiation activity, whereas gray matter NG2 glia are more closely associated with neuronal network activity and synaptic or extrasynaptic signaling. These categories are not mutually exclusive, but together illustrate that NG2 glial heterogeneity under physiological conditions reflects anatomical localization, molecular state, and functional specialization.

Importantly, the structural and regional heterogeneity of NG2 glia is closely linked to their functional diversity. Beyond their well-established role as oligodendrocyte precursors, these cells actively participate in neuronal circuit dynamics through direct communication with neurons. The following section outlines how NG2 glia integrate into neural networks under physiological conditions, providing a basis for understanding how these interactions may be altered after SCI.

### 2.1. Interaction of NG2 Glia with Neurons

As a functionally specialized glial population, NG2 glia engage in bidirectional signaling with neurons, including the formation of bona fide synaptic contacts. NG2 glial cells are capable of forming functional synapses with neurons, first observed in the hippocampus [28], and later in the cerebellum and corpus callosum [29]. Communication between neurons and NG2 glia can occur through both synaptic and non-synaptic mechanisms. Synapses between neurons and NG2 glia contain glutamate receptors of the α-amino-3-hydroxy-5-methyl-4-isoxazolepropionic acid (AMPA) and N-methyl-D-aspartate (NMDA) types [30], muscarinic acetylcholine receptors [31] and γ-aminobutyric acid (GABA) receptors [32]. NG2 glia receive synaptic input from both excitatory and inhibitory neurons, thereby modulating neuronal network activity [33,34]. In addition, NG2 glia synthesize neuromodulatory molecules, such as prostaglandins and neuronal pentraxin 2, and express the key postsynaptic density protein 95 (PSD-95) [10,35]. Although most of these synaptic interactions have been characterized in the brain, they suggest fundamental mechanisms by which NG2 glia may respond to neuronal activity in other CNS regions, including the spinal cord. However, direct evidence for bona fide functional synapses involving NG2 glia in the spinal cord is still lacking.

NG2 glia are capable of responding to both vesicular and non-vesicular synaptic transmission and can detect quantal neurotransmitter release events from nearby neurons or unmyelinated axons [36]. This suggests that NG2 glia may possess properties previously thought to be unique to neurons and exert a more significant influence on neuronal networks [37].

NG2 glia respond indirectly to neuronal activity through AMPARs, NMDARs, and GABARs, thereby regulating oligodendrocyte survival, migration, proliferation, differentiation, and axonal myelination. However, the expression levels and functions of these receptors vary. AMPARs are highly expressed in NG2 glia but decrease markedly (~12-fold) upon differentiation into mature oligodendrocytes [25]. There is compelling evidence that AMPAR activation suppresses NG2 glial proliferation, whereas blocking their activity disrupts both differentiation and proliferation of these cells [38,39]. These activity-dependent mechanisms may be particularly relevant after SCI, where extracellular glutamate levels and neuronal signaling are profoundly altered.

The effect of the transcription factor Neurod1 on the direct differentiation of glial cells into neurons in vitro has been analyzed, along with its primary mechanism of action. It is proposed that high expression of Neurod1 may promote the differentiation of NG2 glia into various neuronal subtypes, including GABAergic, dopaminergic, and cholinergic neurons, through activation of the MAPK pathway [40].

NG2/CSPG4 expressed by NG2 glia regulates neuronal network architecture through secretase activity [10]. Cleavage of NG2/CSPG4 by the α-secretase ADAM10 generates an ectodomain that accumulates in the ECM. The remaining C-terminal fragment undergoes further processing by γ-secretase, resulting in the release of the intracellular domain. This process is important for the regulation of synaptic activity, since the NG2/CSPG4 ectodomain accumulating in the ECM can interact with its components, consistent with our immunoelectron microscopy data demonstrating the presence of NG2/CSPG4 in the ECM in a rat SCI model [41]. We also observed NG2/CSPG4 immunoreactivity not only on the cell membrane but also intracellularly and in myelin membranes, suggesting a complex role of this proteoglycan in intercellular interactions. Together, these molecular and synaptic mechanisms indicate that NG2 glia act as active integrators within the neural microenvironment rather than passive progenitors. Their ability to sense neuronal activity, interact with the ECM, and modulate local circuit function suggests that they participate in broader intercellular communication networks. These properties provide a functional basis for understanding their role in injury, including SCI.

### 2.2. Interaction of NG2 Glia with Astrocytes and Microglia

Although NG2 glial cells have classically been regarded as oligodendrocyte precursors, growing evidence indicates their involvement in complex interactions with other glial cells, including functional interactions with astrocytes and microglia even under physiological conditions. For instance, astrocytes derived in vitro from induced pluripotent stem cells secrete tissue inhibitor of metalloproteinase-1 (TIMP-1), which promotes oligodendrocyte differentiation. Conditioned medium from such astrocytes supports the differentiation of NG2 glia into mature oligodendrocytes both in vitro and in vivo [42].

NG2 glia and astrocytes form close contacts within CNS tissues, including the spinal cord. In the adult spinal cord, NG2 glia and astrocytes are evenly distributed across both white and gray matter; NG2 cells are predominantly localized near blood vessels and synaptic zones, whereas astrocytes are situated closer to neuronal somata and synaptic terminals. This spatial organization creates favorable conditions for interactions and the exchange of signaling molecules within the framework of the neuroglial functional unit [43]. Notably, our previous work demonstrated NG2/CSPG4 immunoreactivity in a subset of astrocytes in the intact spinal cord [41]. Whether this expression reflects lineage relationships, i.e., NG2-glia-derived progeny or represents a distinct astrocytic subpopulation remains unclear. This observation further underscores the molecular overlap and potential bidirectional communication between NG2 glia and astrocytes.

Electrophysiological studies on mouse brains demonstrate that NG2 glial cells express receptors for neurotransmitters, including glutamate and ATP, similarly to astrocytes [44]. Although this expression pattern suggests the potential involvement of NG2 glia in the formation of tripartite synapses, direct evidence under SCI conditions is still lacking, and current observations are limited to synapse-like contacts with axonal endings. Moreover, recordings of calcium signaling in NG2 glia and astrocytes in response to synaptic activity support their functional interaction within glial networks. Astrocytes can influence NG2 glial behavior through the release of trophic factors [45,46,47,48]. Among these, FGF2 is known to promote OPC proliferation and maintenance of the progenitor state, whereas the role of other growth factors such as EGF appears to be more context-dependent and less well defined in this setting. Notably, NG2 glia are the only glial cell type known to form functional synapses with neurons—predominantly excitatory synapses with glutamate as the neurotransmitter. It has been suggested that activation of these synapses may indirectly modulate astrocytic activity through neurotransmitter diffusion and subsequent activation of glial networks in mouse brain [44].

Under normal conditions, microglia maintain tissue homeostasis by “surveying” the state of neurons and glial cells. Evidence indicates the presence of molecular cross-regulation between microglia and NG2 glia, including the production of cytokines and growth factors such as TGF-β and IGF-1 by microglia, which may influence the proliferative and differentiation activity of NG2 cells [49]. In the brain, microglia also participate in the “selection” of NG2 cells during microenvironmental remodeling, supporting their stable distribution and integration into the tissue through the phagocytosis of excess or abnormally functioning cells at early developmental stages or during aging [50].

Thus, under physiological conditions, NG2 glia are actively engaged in intercellular interactions with astrocytes and microglia. These interactions include the exchange of trophic factors, coordination of responses to neuronal activity, and maintenance of tissue homeostasis. Importantly, these baseline interactions provide a framework for understanding how NG2 glia participate in glial crosstalk and tissue remodeling after SCI.

## 3. NG2 Glia in Spinal Cord Injury: Reactive States and Cellular Crosstalk

### 3.1. Temporal Dynamics of NG2 Glial Responses After Spinal Cord Injury

SCI should be considered not as a single static pathological condition, but as a temporally evolving process in which the cellular and extracellular microenvironment changes substantially over time. The acute phase is characterized by primary mechanical tissue disruption followed by early secondary events, including vascular damage, blood–spinal cord barrier disruption, hemorrhage, excitotoxicity, edema, oxidative stress, and initiation of inflammatory signaling [51,52]. In this early period, NG2 glia are exposed to rapidly changing injury-associated signals, including altered neurotransmitter levels, inflammatory mediators, and damage-associated molecular patterns. Consistent with this, NG2 glia have been reported to increase their proliferation after SCI and to accumulate around the lesion site, although their functional contribution at this stage may include both reparative and scar-associated responses [53].

During the subacute phase, which generally extends from several days to weeks after injury, the lesion environment becomes increasingly organized into distinct cellular and extracellular compartments, including the lesion core, glial border, and surrounding reactive parenchyma. This period is associated with ongoing inflammatory cell recruitment, astrocytic and microglial reactivity, extracellular matrix remodeling, demyelination, and attempts at remyelination [54]. Within this context, NG2 glia may contribute to repair by generating oligodendrocyte lineage cells involved in remyelination of spared axons. At the same time, reactive NG2 glia are also incorporated into the glial scar, where they may upregulate NG2/CSPG4 and other scar-associated molecules that are linked to inhibition of axonal growth [6,53]. Therefore, the subacute phase appears to be a critical period in which NG2 glial responses may shift the balance either toward remyelination and tissue stabilization or toward the formation of a growth-inhibitory extracellular matrix.

In the chronic phase, the lesion site becomes more structurally stabilized, but the injured spinal cord remains biologically active. Chronic SCI is associated with persistent tissue remodeling, incomplete remyelination, axonal dieback, cystic cavity formation, and long-term changes in the inflammatory and extracellular matrix environment [54,55]. In this setting, NG2 glia may persist within or around the scar and maintain context-dependent functions. On the one hand, they represent a resident progenitor population with potential relevance for remyelination. On the other hand, NG2/CSPG4-positive cells and NG2/CSPG4-rich matrix components have been associated with dystrophic axon stabilization and the formation of synapse-like axonal “traps”, which may contribute to regeneration failure [4]. Thus, the functional meaning of NG2 glial activation depends strongly on the phase of SCI, the local tissue compartment, and the balance between reparative and inhibitory signals.

A stage-dependent view is therefore essential for interpreting NG2 glial biology after SCI. The same cellular processes, including proliferation, migration, cytokine responsiveness, NG2/CSPG4 expression, extracellular matrix remodeling, and interactions with injured axons, astrocytes, microglia/macrophages, and vascular elements, may have different consequences during acute, subacute, and chronic stages. The main phase-specific roles of NG2 glia after SCI are summarized in Table 1.

### 3.2. Reactive NG2 Glia in the Injured Spinal Cord: Tissue Context, Plasticity, and Inflammatory Signaling

Following SCI, the tightly regulated physiological interactions involving NG2 glia become profoundly altered, as these cells are exposed to inflammation, tissue disruption, and extensive ECM remodeling. Under these conditions, NG2 glia acquire reactive phenotypes and contribute to both reparative and inhibitory processes within the injured spinal cord.

After SCI, a complex tissue structure forms at the lesion site, comprising both fibrotic and glial scars, as well as the adjacent reactive neural parenchyma with unique cellular and molecular compositions [56,57]. The fibrotic scar, located in the lesion epicenter, consists of pericytes, fibroblasts, and endothelial cells, and is characterized by a high density of collagen I and other extracellular matrix components that inhibit axonal growth. NG2^+^ perivascular cells within the fibrotic scar represent a vascular-associated population expressing PDGFRβ and NG2/CSPG4. These cells have been implicated in angiogenesis and fibrotic tissue organization, including contributions to collagen-rich matrix deposition, although collagen I production is not restricted to NG2^+^ cells [18].

The glial scar consists of reactive astrocytes, microglia, and NG2 glia, as well as the adjacent reactive neural parenchyma, which differs in its reduced reactivity and the presence of neurons undergoing active synaptic remodeling and circuit reorganization [58,59,60]. Within both the glial scar and adjacent reactive parenchyma, NG2 glia after SCI actively proliferate, exhibit process retraction, and undergo hypertrophy.

These cells accumulate at the lesion site, where they serve a dual role: contributing to remyelination while simultaneously inhibiting axonal growth through the production of inhibitory molecules, including NG2/CSPG4 [53,61]. Recent single-cell transcriptomic studies indicate that glial progenitor responses after SCI are heterogeneous and cannot be reduced to a uniform reactive OPC/NG2 glial state. Importantly, this heterogeneity should be interpreted not only as a baseline regional and functional property of NG2 glia in the intact CNS, but also as an injury-associated and spatially dependent phenomenon. In a single-cell RNA sequencing analysis of mouse SCI, glial progenitor populations showed injury-associated heterogeneity, including cell states linked to proliferation, inflammatory responsiveness, and oligodendrocyte lineage progression [62]. These data are consistent with the concept that post-SCI NG2 glial states may differ according to their spatial location, lesion compartment, interaction partners, and exposure to inflammatory, vascular, axonal, or extracellular matrix cues.

Spatially resolved transcriptomic studies of the CNS, including the spinal cord, further support the idea that oligodendrocyte lineage progression is regionally heterogeneous and that the timing of differentiation and myelination may vary across anatomical compartments [63]. In the SCI context, such spatial heterogeneity is particularly important because NG2 glia located in the lesion core, glial border, perilesional white matter, or more remote spinal segments are likely exposed to different combinations of cytokines, axonal signals, vascular cues, and extracellular matrix components. Therefore, single-cell and spatial transcriptomic data suggest that the functional role of NG2 glia after SCI depends not only on their lineage identity, but also on their injury-induced state and anatomical microenvironment. Future studies combining lineage tracing, spatial transcriptomics, and functional assays will be necessary to determine which NG2 glial subpopulations support remyelination and which contribute to scar-associated inhibition or chronic tissue remodeling.

Another studies, including fate mapping, have demonstrated that under SCI conditions, NG2 glia can differentiate not only into oligodendrocytes but also into astrocytes expressing anti-inflammatory and anti-apoptotic proteins, thereby contributing to the formation of a heterogeneous population within the glial scar [17,64,65,66]. During the establishment of the glial scar boundary, Wnt signaling regulates the multipotent differentiation of NG2 glia into astrocytes and oligodendrocytes, whereas their differentiation into reactive astrocytes is driven by Shh signaling [67]. The proportion of NG2 glia differentiating into astrocytes within the glial scar and reactive parenchyma varies depending on the type of injury: in a contusion SCI model, 25% of NG2 glia progeny became GFAP^+^ cells [17], whereas in a partial dorsal funiculus transection model their proportion was only 5% [19].

It should also be noted that NG2 glia can participate in the regulation of inflammation through the expression of receptors for IL-6, IL-10, TNF-α, and other cytokines, activation of NF-κB, and the expression of MHC-II molecules, suggesting their potential role as antigen-presenting cells [66]. The alarmin HMGB1 (high-mobility group box-1), a biomarker of neuroinflammation released by cortical neural stem cells (NSCs), has been shown to exert antiproliferative and pro-reactive effects on NG2 glia in an in vitro scratch model of traumatic brain injury (TBI) [68]. A reduction in NG2 glial proliferative activity has also been reported within the glial scar after SCI in knockout mice lacking β-catenin, confirming the key role of the Wnt/β-catenin signaling pathway in mediating this effect [69].

In response to SCI, NG2 glia become activated and actively migrate to the lesion site, guided by elevated glutamate levels. One of the earliest and most significant changes in the microenvironment after SCI is a sharp increase in extracellular glutamate, the principal excitatory neurotransmitter in the CNS. This rise is associated with neuronal membrane disruption, impaired glutamate transport, and glial cell activation, and it contributes to glutamate-mediated excitotoxicity that causes additional neuronal death. At the same time, glutamate also serves a signaling role by specifically activating NG2 glia, which express both ionotropic (AMPA, NMDA) and metabotropic (mGluR) glutamate receptors [70].

In addition to neuronal sources, glutamatergic signaling to NG2 glia can also be mediated by astrocytes. In vitro studies have shown that glutamate released via connexin 43 (Cx43) in astrocytes can suppress NG2 glial differentiation through the activation of ionotropic glutamate receptors—AMPA and NMDA. Blocking Cx43 reduces glutamate secretion and promotes NG2 glial maturation [71]. Although Cx43 itself is unlikely to represent a therapeutic target due to its widespread expression and systemic functions, these findings underscore the importance of local glutamatergic regulation of NG2 glia via AMPA/NMDA receptors as a potential point of intervention.

Together, these findings indicate that, beyond responding to neurotransmitters and inflammatory mediators, reactive NG2 glia undergo a profound shift in their effector functions after SCI. In particular, activation of NG2 glia is accompanied by marked changes in ECM production and remodeling, positioning these cells as key regulators of the post-traumatic tissue microenvironment. This transition from signal sensing to structural remodeling is largely mediated by NG2/CSPG4 and other ECM-associated molecules, which critically influence axonal growth and regenerative failure.

### 3.3. NG2/CSPG4-Dependent Extracellular Matrix Remodeling and Inhibition of Axonal Regeneration

Reactive NG2 glia play an important role in secreting ECM components, such as CSPGs, heparan sulfate proteoglycans (HSPGs), glycoproteins, and others, some of which stabilize the lesion site and prevent its spread, while simultaneously inhibiting axonal growth [72,73]. Key molecular hallmarks of the reactive state of NG2 glia include the expression of the proliferative marker Ki67, absent under physiological conditions [74], as well as a sharp increase in the production of NG2/CSPG4, which contributes to glial scar formation and axonal growth inhibition [53,61]. NG2/CSPG4, like other CSPGs, interacts with receptors on axonal membranes, including PTPσ and LAR, thereby triggering signaling cascades that suppress axonal growth [75,76]. Suppression of NG2/CSPG4 using AAV vectors expressing scFv antibodies (single-chain variable fragment) against NG2/CSPG4 has been shown to improve synaptic transmission, remyelination, and motor function recovery in rats after SCI [77]. In addition, reactive NG2 cells are associated with the deposition or local enrichment of several inhibitory ECM components, including lectican family CSPGs and tenascins, although these molecules are also produced by astrocytes and other cell types within the lesion environment. After SCI, the expression of aggrecan, versican, neurocan, brevican, phosphacan, and tenascin C increases markedly in the scar region, where they contribute to tissue remodeling and inhibition of axonal growth. HSPGs such as syndecan 3 and the associated enzyme heparanase are also involved in ECM remodeling and cell interactions after injury [6,13,61,78,79,80].

An important factor determining the balance between proliferation and differentiation of NG2 glia is the proteolytic cleavage of NG2/CSPG4. Transmembrane NG2/CSPG4 has been shown to enhance PDGFRα signaling by binding PDGF-AA and maintaining the proliferative status of NG2 cells. During differentiation of NG2 glia into oligodendrocytes, the expression of the metalloproteinase ADAMTS-4 increases, leading to shedding of NG2/CSPG4 and a reduction in its surface levels. This, in turn, attenuates PDGFRα-mediated activation of ERK signaling and promotes differentiation and remyelination. ADAMTS-4 deficiency is associated with the accumulation of NG2/CSPG4 on the membrane, enhanced PDGFRα signaling, and delayed oligodendrocyte maturation, ultimately impairing myelin repair after lysophosphatidylcholine-induced demyelination in mice [81]. In addition, neutrophils, which are predominantly activated after SCI, secrete MMPs that remodel the ECM, among which MMP-9 cleaves NG2/CSPG4 into its active form. This active or shed form is involved in the formation of both fibrotic and glial scars [82,83]. At the same time, the soluble fragment of NG2/CSPG4 generated after shedding exerts an inhibitory effect on axons and contributes to the formation of so-called “traps” for dystrophic axons, leading to growth termination and the establishment of synapse-like contacts at the outer border of the glial scar after SCI [4,73,82]. These findings highlight the dual role of NG2/CSPG4 as both a marker and a functional fate regulator of NG2 glia and point to proteolytic cleavage of NG2/CSPG4 as a key mechanism in post-traumatic events.

In addition to NG2 glia, NG2/CSPG4 expression under pathological conditions has been reported in reactive astrocytes, macrophages, pericytes, and endothelial cells [18,84]. Following SCI, NG2/CSPG4 levels in the glial scar area rise sharply during the first 7 days, primarily due to NG2 glia and reactive astrocytes [18], and, albeit to a much lesser extent, expression in these cells may persist into the chronic phase [41]. Further insights into the cellular sources of NG2/CSPG4 were provided in our subsequent work [85], which demonstrated that in the chronic phase of SCI, the number of NG2 glia does not correlate with NG2/CSPG4 expression in either the perilesion area or more distant regions. Importantly, our data also indicate that microglia contribute minimally to NG2/CSPG4 expression, both at the lesion site and in remote areas (unpublished data). Conflicting reports exist regarding whether pericytes, which exhibit bona fide NG2/CSPG4 expression, can differentiate into microglia in the brain after stroke [86]; however, this notion was later convincingly refuted [87]. Nevertheless, in acute brain injuries, microglia have been shown to exhibit NG2/CSPG4 expression in a disease-dependent manner [87]. This cellular heterogeneity in NG2/CSPG4 sources may have functional significance, as expression patterns across different cell types determine its availability for interactions with neuronal networks and ECM components.

Wennström et al. (2014) [88] demonstrated that systemic inflammation induced by LPS, as well as proinflammatory cytokines (IL-1β and IL-6), markedly enhance proteolytic shedding of the extracellular domain of NG2/CSPG4 both in vivo in rats and in vitro in human brain OPC cultures. This is accompanied by reduced proliferation of NG2 glia and elevated levels of the soluble form of NG2/CSPG4 (sNG2) in the extracellular environment, including cerebrospinal fluid [88]. Proteolytic shedding of NG2/CSPG4 has been shown in vitro [89] and, for MMP-14, in vivo in a rat sciatic nerve injury model [90]. Thus, NG2 labeling within the glial scar may partly reflect the accumulation of shed ectodomain fragments in the ECM [91], although direct evidence for this mechanism in the injured spinal cord is still lacking. These findings suggest that the post-injury inflammatory milieu promotes accumulation of sNG2-a fragment capable of shaping an inhibitory microenvironment and potentially forming adhesive “traps” for axons. Such properties make NG2/CSPG4 one of the central molecular inhibitors of axonal growth after SCI.

It can be assumed that increased expression of NG2/CSPG4 in reactive glia at the lesion site enhances shedding of the extracellular domain (ectodomain), mediated by the α-secretase ADAM10, thereby amplifying its physiological effects [10,92]. Despite the well-established role of NG2/CSPG4 in ECM regulation and glial scar formation, the differential effects of its transmembrane and soluble (shed) forms remain poorly understood. Available data suggest that these forms may serve distinct functions: for example, the soluble form, once released into the ECM, contributes to ECM remodeling and may enhance axonal growth inhibition, whereas the membrane-associated form participates in local barrier formation and cell–cell interactions [73,93]. However, the cell-type-specific effects of transmembrane and sNG2 forms on neurons, astrocytes, microglia, and oligodendrocytes remain unclear, as systematic comparative studies are lacking. This gap is particularly critical given that structurally related molecules, such as syndecans and other proteoglycans, have already been shown to exert distinct functions depending on whether they are membrane-associated or shed-the latter forms being involved in calcium homeostasis, synaptic synchronization, and cellular differentiation [94]. This suggests that similar functional differences may exist for NG2/CSPG4. Moreover, CSPGs exhibit a wide spectrum of effects on synaptic function: they are integral components of perineuronal nets and stabilize mature synapses, thereby limiting neuroplasticity once critical developmental periods have ended [95,96]. In addition, certain CSPGs, including NG2/CSPG4, can directly reduce axonal conduction, as demonstrated in spinal cord models with exogenous administration [97]. Despite the predominantly inhibitory effects of CSPGs, under some conditions they can bind neurotrophic factors (e.g., BDNF, Midkine), supporting neuronal growth and survival. However, the influence of specific CSPG forms—and particularly NG2/CSPG4—on synapse formation, stabilization, and function in diverse neuronal contexts, including potential contacts with NG2 glia, remains unresolved, underscoring the need for further comprehensive studies in this area.

Several studies have shown that reactive NG2^+^ cells produce chemokines and cytokines that promote the recruitment and activation of microglia. NG2 glia are also associated with the blood–brain barrier (BBB), supporting its integrity through TGF-β-dependent signaling. Inhibition of TGF-β secretion by NG2 glia has been reported to cause extensive cerebral vascular ruptures and hemorrhages. Moreover, NG2 glia enhance the expression of tight junction proteins that connect endothelial cells via activation of the TGF-β signaling pathway, thereby further strengthening BBB integrity [98].

Thus, NG2 glial reactivity represents a specialized state with a unique molecular profile and a dual function—on the one hand supporting repair, while on the other restricting axonal regeneration through the production of inhibitory molecules. Our previous findings of a gradient of NG2/CSPG4 and Olig2 expression in the ventral horns after contusive SCI in rats further confirm the existence of spatially dependent remodeling of the glial environment. Such remodeling may either support or hinder neuroregeneration depending on the distance from the lesion epicenter. In addition, our data indicate that the increase in reactive NG2^+^ cells occurs predominantly in close proximity to the epicenter of injury during the early post-SCI period [41,85]. Importantly, this spatially restricted reactivity places NG2 glia in direct contact with injured neurons, positioning neuron–NG2 glia communication as a critical component of post-traumatic remodeling.

### 3.4. Neuron–NG2 Glia Communication: Synaptic and Paracrine Mechanisms

One of the earliest and most functionally relevant aspects of NG2 glial reactivity after SCI is their interaction with damaged neurons. In addition to their involvement in remyelination and glial scar formation, NG2 glia establish both direct and indirect interactions with injured neurons. Within the first days after SCI, a marked increase in NG2^+^ cells is observed in the perilesion area, where they are located in close proximity to damaged neurons [15,85]. This localization suggests a directed response of NG2 glia to signals from dying or degenerating neurons. The ability of NG2 glia to form synapse—like contacts with neurons and to receive glutamatergic input-displaying properties analogous to neuronal synapses, including rapid and quantal responses, potentiation, and depression—has been demonstrated in the hippocampus, but has not yet been studied in the spinal cord [28,70,99]. Additionally, it has been shown that in the cerebellum NG2 glia are capable of forming functional synaptic contacts due to elevated expression of AMPA receptors [100]. Although this has not yet been directly demonstrated in the spinal cord, available data suggest that NG2 glia may respond to local increases in glutamate and contribute to sensing injury-related changes in the microenvironment, which in turn may influence neuronal network organization after SCI.

Under normal conditions, NG2 glia participate in synaptic activity by receiving excitatory input from neurons. However, after SCI, the functional relationship between neurons and NG2 glia undergoes significant changes. As noted earlier, NG2 glial contact with axons leads to the formation of synapse-like structures. These contacts are accompanied by the local accumulation of synaptic markers such as synaptic vesicle protein 2 (SV2) and synaptosomal-associated protein 25 (SNAP-25), but they do not mediate functional synaptic transmission [4]. Thus, instead of contributing to synaptic network restoration, NG2 glia in the context of injury establish non-communicative contacts that restrict neuronal plasticity. Given that the formation of these contacts is mediated by interactions between the NG2 glial cell surface and extracellular components, including CSPGs and adhesion molecules, their emergence may represent a specific molecular mechanism of regeneration inhibition rather than merely a structural consequence of trauma. In this regard, a promising direction for future research lies in the detailed characterization of these contact zones, including mediator phenotypes, synaptic markers, and electrophysiological evidence in the penumbra after SCI, in order to identify key molecules responsible for their stabilization and to develop strategies for their selective destabilization. Such approaches may open new opportunities for promoting axonal growth and reinnervation of damaged CNS regions.

Following injury, NG2 glia continue to respond to neurotransmitters, including glutamate, but their sensitivity and responses to neuronal activity are altered. In particular, after contusive SCI in rats, NG2 glia in the lesion epicenter exhibit reduced amplitude and frequency of AMPAR-mediated currents, decreased density of voltage-gated potassium currents, and altered expression of transcription factors such as Olig2 and Sox10, indicating a transition to a different functional state [101]. These changes reflect an adaptation of NG2 glia to the evolving microenvironment and a potential shift toward a regeneration-associated phenotype.

One of the key roles of NG2 glia after SCI is the restoration of myelin sheaths around damaged axons. NG2^+^ cells are capable of differentiating into oligodendrocytes and contributing to the remyelination of surviving neuronal processes, thereby supporting their survival and conduction [53,102]. However, the efficiency of remyelination depends on both axonal integrity and the local microenvironment, including inflammatory mediators and inhibitory molecules that can limit the differentiation of NG2 glia into mature, functional oligodendrocytes [103,104,105].

Remyelination after SCI depends not only on the availability and intrinsic differentiation capacity of NG2 glia, but also on the state of denuded or spared axons and the molecular signals they provide. In experimental demyelination models, electrically active demyelinated axons can form de novo synaptic contacts with recruited OPCs, and neuronal activity-dependent glutamate release has been shown to promote OPC differentiation into myelinating oligodendrocytes [106]. Although these findings were obtained in demyelinating lesions rather than in traumatic SCI, they are relevant for interpreting post-SCI remyelination because spared axons in the injured spinal cord represent the substrate on which newly differentiated oligodendrocytes must form myelin sheaths.

In addition to activity-dependent signaling, contact-mediated axon-glia interactions are important regulators of myelination and remyelination. Several axonal surface-associated molecules can either permit or restrict oligodendrocyte differentiation and myelin formation. For example, polysialylated neural cell adhesion molecule (PSA-NCAM), which is enriched on non-myelinated or demyelinated axons, has been shown to negatively regulate CNS myelination. Its persistence on the axonal surface may therefore create a non-permissive environment for myelin formation [107,108]. Another example is axonal LINGO-1: nerve growth factor was shown to increase axonal LINGO-1 expression, and axonal LINGO-1 inhibited oligodendrocyte differentiation and myelination in vitro and in vivo [109]. These mechanisms indicate that axons are not passive substrates for remyelination, but actively regulate whether recruited OPCs proceed toward mature myelinating oligodendrocytes.

This concept is particularly relevant after SCI, where surviving axons are exposed to inflammatory mediators, altered extracellular matrix composition, and scar-associated inhibitory molecules. Under these conditions, axon-derived or axon-associated signals may become uncoupled from the differentiation program of NG2 glia/OPCs. In parallel, NG2/CSPG4-rich extracellular matrix components and synapse-like contacts between NG2 glia and dystrophic axons may stabilize non-productive axon-glia interactions rather than support remyelination and axonal extension [4]. Thus, remyelination failure after SCI may reflect not only insufficient OPC recruitment or differentiation, but also the presence of axonal surface and extracellular cues that limit productive axon-OPC communication.

The neuronal differentiation potential of NG2 glia remains a matter of debate and should be interpreted according to the experimental context. Under physiological conditions, genetic fate-mapping studies generally indicate that NG2 glia remain largely committed to the oligodendrocyte lineage and do not normally generate neurons in the adult CNS or spinal cord [110,111,112]. In this respect, evidence for neuronal conversion should be distinguished from the well-established ability of NG2 glia to generate oligodendrocytes and contribute to remyelination.

At the same time, several experimental studies suggest that NG2 glia can acquire broader plasticity under defined conditions. In vitro studies have shown that NG2 glia may adopt neural stem cell-like or neuronal phenotypes in response to specific transcriptional or environmental cues. For example, NeuroD1 overexpression has been proposed to promote neuronal differentiation programs in glial cells, although such findings should be interpreted as induced reprogramming rather than evidence of spontaneous neuronal differentiation in vivo. Similarly, in vivo reprogramming approaches have suggested that the post-injury spinal cord environment may reveal a latent neurogenic potential of NG2 glia when combined with forced expression of neurogenic factors such as SOX2 [113]. These data are important because they demonstrate that NG2 glia can be experimentally redirected toward neuronal phenotypes under specific conditions.

However, definitive evidence that endogenous NG2 glia spontaneously generate functionally integrated neurons after SCI remains limited. Reports of neuronal marker expression or neuronal-like phenotypes should therefore be separated from lineage-traced neuronal conversion and from functional evidence of mature neuronal integration into spinal circuits. Thus, in the context of SCI, NG2 glia should primarily be considered oligodendrocyte lineage cells with context-dependent plasticity, while their neuronal differentiation potential represents an experimentally inducible phenomenon that requires careful validation using lineage tracing, electrophysiological characterization, and functional integration analyses.

Taken together, interactions between NG2 glia and damaged neurons after SCI reveal a pronounced functional dualism: while providing remyelination and trophic support, NG2 glia simultaneously restrict regeneration through scar formation and the secretion of growth-inhibitory molecules. Importantly, these neuron-directed effects do not occur in isolation but are embedded within broader glial interaction networks, suggesting that NG2 glia operate at the interface of multiple cellular responses after injury.

### 3.5. NG2 Glia in Glial Crosstalk: Shaping Astrocytic and Microglial Responses

After SCI, a complex cascade of cellular and molecular responses is initiated, aimed at limiting tissue damage and promoting repair, while simultaneously driving inflammatory processes that can impair regeneration. Within this context, NG2 glia, astrocytes, and microglia form an interconnected signaling network that critically shapes the post-injury microenvironment. Beyond their individual contributions to glial scar formation and myelin repair, these cell types engage in reciprocal interactions that influence cellular reactivity, inflammatory tone, and tissue remodeling. Understanding how NG2 glia integrate into and modulate astrocyte–microglia crosstalk is therefore essential for identifying mechanisms that determine injury outcome and therapeutic responsiveness.

Following SCI, there is a marked proliferation of NG2^+^ cells [18,114]. However, they are unable to effectively replace lost cell populations either in the lesion epicenter and adjacent penumbra or, to varying degrees, in more distant regions that also display degenerative changes after SCI. Our studies have shown that remote lumbar segments following thoracic contusion injury exhibit features of adaptive remodeling and chronic inflammation, including alterations in neuronal and glial marker expression, remodeling of the ECM, microglial activation, and disruption of the blood–spinal cord barrier [41,85,115,116,117]. Returning to the lesion epicenter, it can be assumed that the limitation of NG2 glial interactions arises from the inhibitory influence of microglia and macrophages. Indeed, microglia and macrophages secrete factors that suppress the growth of NG2^+^ cells in culture, as demonstrated in experiments using conditioned medium from OX42^+^ microglia. Substances released by activated microglia include TNF-α and MMP-9, which exert inhibitory effects on NG2 glial growth. These findings suggest that the inability of NG2 glia to replace damaged cells in the lesion site may be linked to the actions of such inhibitory factors produced by microglia and macrophages [114].

NG2 glia participate in glial scar formation as part of a coordinated response involving astrocytes, microglia, and NG2 glia, which together shape the inflammatory and structural landscape after SCI. Beyond contributing to remyelination, NG2 glia upregulate CSPGs and integrate into the developing scar border, where their behavior is strongly influenced by astrocyte- and microglia-derived signals. Microglial activation enhances astrocytic STAT3 signaling, which regulates hypertrophy and CSPG deposition, thereby modifying the ECM into which NG2 glia are incorporated. Disruption of this microglia-dependent astrocytic response impairs scar maturation and exacerbates inflammation, thereby altering the lesion microenvironment in which NG2 glia operate after SCI [118]. In parallel, inflammatory cues amplified by the astrocyte-derived fibronectin/β1-integrin pathway increase microglial production of TNF-α and MMPs, factors known to suppress NG2 glial proliferation and shift them toward a more reactive, CSPG-producing phenotype [114,119].

While astrocyte-microglia crosstalk in SCI is well characterized, direct mechanistic interactions linking these pathways to NG2 glia remain poorly defined. Available evidence indicates that NG2 glia respond predominantly to the microenvironment shaped by astrocyte-microglia signaling rather than through direct crosstalk highlighting an important limitation of current knowledge.

Moreover, under conditions of induced brain inflammation, NG2 glia release TGF-β2, which acts on microglia through TGFBR2 and CX3CR1 receptors, modulating their reactivity and suppressing inflammation [91,120]. Conversely, in SCI and under pronounced neuroinflammatory conditions, NG2 glia acquire an immunomodulatory phenotype while losing part of their reparative functions, such as the efficiency of remyelination. In particular, co-culture of NG2^+^ cells with activated microglia (OX42^+^) or exposure to proinflammatory molecules (TNF-α, MMP-9, TSP-1, TIMP-1) suppressed NG2^+^ cell proliferation, indicating that the inflammatory microenvironment exerts an inhibitory effect on the proliferative activity of NG2 glia [114].

Thus, NG2 glia, astrocytes, and microglia form a complex network of interactions after SCI that governs both reparative and inflammatory processes. These cells actively participate in glial scar formation, which simultaneously impedes axonal regeneration and limits the spread of inflammation. Their interactions through signaling pathways such as STAT3 and fibronectin/β1 integrin, as well as inflammatory molecules including CCL2 and IL-1β, may play a key role in injury outcomes and represent potential targets for therapeutic intervention in SCI. Nevertheless, critical aspects of NG2 glia interactions with astrocytes and microglia, including the molecular mechanisms of their communication and the factors that determine the functional plasticity of NG2 cells, remain unclear and require further investigation using advanced methods of intercellular interaction analysis.

To avoid considering these interactions as isolated cell-to-cell events, NG2 glial crosstalk after SCI can be viewed through several overlapping functional axes. The first is an inflammatory axis, in which NG2 glia respond to cytokines, microglia/macrophage-derived mediators, and astrocyte-dependent inflammatory signals. The second is a regenerative and remyelinating axis, in which NG2 glia interact with spared axons and oligodendrocyte lineage cells and may contribute to remyelination. The third is an ECM and scar-remodeling axis, in which NG2/CSPG4 and other proteoglycan-associated mechanisms participate in the formation of a growth-inhibitory environment. Finally, a vascular and metabolic-support axis may involve interactions with endothelial and perivascular cells, including mechanisms related to blood–spinal cord barrier integrity. These axes are not independent; rather, they converge within the lesion border and perilesional tissue, where NG2 glia may simultaneously support repair and contribute to regeneration-limiting scar properties.

**Figure 1:**
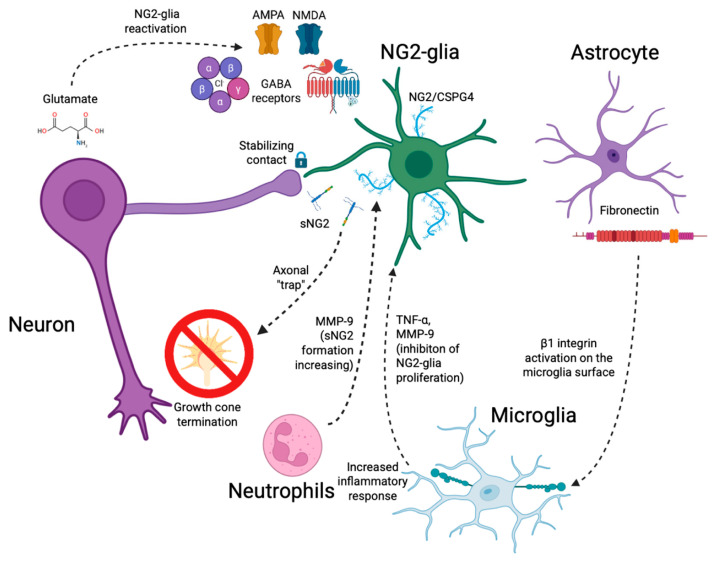
Integrated NG2 glia-centered cellular crosstalk in the perilesional microenvironment after spinal cord injury (SCI). The scheme summarizes experimentally supported and proposed interactions involving NG2 glia after SCI. Some of these mechanisms have been directly demonstrated in the spinal cord, whereas others are inferred from related CNS contexts and are shown here as putative interactions. Following SCI, NG2 glia become reactive and respond to changes in the local microenvironment, including elevated extracellular glutamate and inflammatory mediators. Activation of ionotropic AMPA and NMDA receptors, as well as GABA receptors, is suggested to contribute to NG2 glial reactivity. Reactive NG2 glia upregulate NG2/CSPG4 and participate in extracellular matrix remodeling at the lesion border. NG2/CSPG4 may undergo proteolytic shedding, generating a soluble form, sNG2, a process associated with neutrophil-derived MMP 9 and increased accumulation of sNG2 in the extracellular matrix. These changes are linked to the formation of a growth inhibitory microenvironment and stabilization of dystrophic axonal endings, or axonal traps, limiting further axonal extension. In parallel, astrocyte-derived fibronectin may activate β1 integrins on microglia, enhancing inflammatory signaling and promoting the release of factors such as TNF α and MMP 9, which may indirectly influence NG2 glial behavior.

In summary, regarding cellular crosstalk as a whole, it becomes evident that NG2 glia do not exist in isolation but are integrated into a complex network of intercellular interactions after SCI. These interactions encompass both remyelinating and neuroprotective mechanisms, as well as processes associated with scar formation and axonal growth inhibition. Some of these mechanisms have been characterized primarily in the brain or under experimental gain of function conditions, and their physiological relevance in the adult spinal cord remains to be established. The functional outcomes of NG2 glia interactions with neurons, astrocytes, microglia, and endothelial cells are summarized in Table 2. An integrated NG2 glia-centered overview of experimentally supported and proposed interactions within the SCI microenvironment is presented in Figure 1. These data highlight the dual role of NG2 glia and provide a foundation for developing potential therapeutic strategies, which will be discussed in the following section.

## 4. Potential Therapeutic Strategies

NG2 glia and the proteoglycan NG2/CSPG4 they express occupy a central position in CNS homeostasis by supporting oligodendrogenesis, modulating neuronal activity and synaptic plasticity, and regulating cell migration, ECM organization, and axonal growth. As highlighted in the previous sections, these functions place NG2 glia at the intersection of reparative and inhibitory processes following SCI. Importantly, this dual role renders NG2 glia not only contributors to post-traumatic pathology but also attractive therapeutic targets. Accordingly, strategies aimed at modulating NG2 glial activity and NG2/CSPG4-dependent signaling may offer new opportunities to enhance remyelination, restore neuronal connectivity, and promote functional recovery after SCI.

After CNS demyelination, OPCs initiate remyelination by undergoing sequential stages of activation, migration, proliferation, and differentiation into mature oligodendrocytes. This process is regulated by both intrinsic cellular mechanisms and extrinsic microenvironmental signals. According to Huntemer-Silveira et al. (2021) [105], the remyelination potential of OPCs declines with age, partly due to reduced expression of differentiation factors and mitochondrial dysfunction. In addition, the inflammatory microenvironment-including TNF-α, IFN-γ, and TGF-β-activates Notch, Wnt, and BMP signaling pathways, thereby disrupting OPC maturation [103]. Effective stimulation of remyelination thus requires overcoming these inhibitory influences and restoring OPC responsiveness to regenerative cues [103,105]. Molecular regulators such as Myt1L, PPARγ, and Tet3 positively modulate the expression of myelin-related genes, whereas TIP30, RhoA/ROCK, and BMP exert inhibitory effects. Therapeutic strategies under consideration include modulation of these pathways and pharmacological “rejuvenation” of OPCs-for example, by metformin treatment or fasting-which can partially restore their differentiation potential [121,122].

A study by Whittaker et al. (2012) focused on the use of the glial growth factor GGF2 to enhance NG2 glial proliferation [123]. It was demonstrated that GGF2 activates signaling pathways responsible for NG2 glial proliferation and differentiation into oligodendrocytes. The resulting increase in oligodendrocyte numbers promoted remyelination, which in turn improved spinal cord function after injury. Furthermore, treatment with GGF2 was shown to upregulate Sox2, a key stem cell regulator, thereby supporting the maintenance of stem-like properties in NG2 glia [123].

A gene therapy-based approach has also been explored, aiming to reduce NG2/CSPG4 expression in glial cells (including NG2 glia) in combination with the application of neurotrophin-3 (NT-3). This strategy demonstrated efficacy in reducing scar size at the injury site and improving motor function in mice subjected to SCI. The combination of NG2/CSPG4 downregulation and NT-3 administration led to a significant enhancement of regenerative processes [124].

NG2 glia represent a promising therapeutic target for SCI, traumatic brain injury, multiple sclerosis, and other demyelinating conditions due to their constant presence in all neurogenic niches of the central nervous system. Genetic factors such as Sox2, Olig2, Pax6, and PDGFRα are markedly overexpressed in NG2 glial populations, promoting gliogenesis and the activation of stem-like properties [125]. However, neurogenesis in the post-traumatic CNS is profoundly impaired, leading to ineffective maintenance of homeostasis as well as deficits in learning and memory, ultimately resulting in dysfunctional neuronal networks.

Apotransferrin also promotes NG2 glial differentiation through the MAPK/ERK1/2 and PI3K/Akt/mTOR signaling pathways, which play a key role in oligodendrocyte maturation [126]. Recent studies have revealed crosstalk between apotransferrin and thyroid hormone during oligodendrocyte differentiation in development [127]. Inactivation of PTEN, an inhibitor of the PI3K/Akt/mTOR cascade, in NG2 glia cultures from both rats and humans induces their maturation into oligodendrocytes when co-cultured with IGF-1 [128]. Genetic ablation of ERK1/2 in NG2 glia disrupts the rate of their differentiation but does not affect proliferation during development [129]. Another study demonstrated a strong correlation between ERK1/2 activation induced by interleukin-17A and NG2 glial differentiation [130]. Inhibition of ERK1/2 signaling by silencing its upstream activator B-Raf impairs NG2 glial differentiation both in cell culture and in vivo. However, another report found no effect of either sustained activation or deletion of ERK1/2 on NG2 glial differentiation [131].

The body of evidence highlights the pivotal role of NG2 glia as a plastic and therapeutically relevant cell population in recovery after spinal cord injury. These cells possess the capacity for proliferation, differentiation, and intercellular communication, and they also demonstrate potential for reprogramming into neurons as well as for exerting neuroprotective functions. Reprogramming mediated by factors such as SOX2, BDNF, and NT-3 opens the possibility of directed generation of functional neurons and the restoration of neuronal circuits. In parallel, stimulation of remyelination through growth factors (e.g., GGF2), signaling pathways (STAT3, PI3K/Akt, ERK1/2), and modulation of NG2/CSPG4 expression can significantly enhance the recovery of conduction and motor function. Together, these strategies form a multilayered framework in which NG2 glia are regarded not only as supportive elements but also as active participants in regeneration, thereby opening avenues for the development of novel therapeutic approaches for spinal cord injury.

## 5. Conclusions

An increasing body of evidence suggests that NG2 glia in SCI act not only as a population of oligodendrocyte progenitors but also as a dynamic hub of cellular crosstalk that shapes the outcome of post-traumatic processes. This review highlights their dual nature: contributing to remyelination and neuroprotection on the one hand, while also participating in glial scar formation and inhibition of axonal regeneration on the other. The cellular crosstalks we have discussed form a complex signaling network, the balance of which determines whether reparative or inhibitory processes predominate. Viewing these events through the lens of cell–cell interactions positions NG2 glia as a key integrator of post-traumatic signaling in SCI. Integration of our own experimental data on spatial NG2/CSPG4 expression and the dynamics of NG2^+^ cells in contusion SCI underscores the heterogeneity of this population and the need to consider regional differences in therapeutic design. Collectively, this frames NG2 glia not only as a subject of fundamental research but also as a promising therapeutic target, where modulation of intercellular interactions may open new avenues for recovery after neurotrauma.

## Figures and Tables

**Table 1:** Stage-dependent roles of NG2 glia after spinal cord injury.

SCI Phase	Approximate Timing	Dominant Pathological Events	NG2 Glial Responses	Major Cellular Interactions	Functional Implications	Source
Acute phase	Hours to several days	Primary tissue disruption, hemorrhage, blood–spinal cord barrier disruption, edema, excitotoxicity, oxidative stress, early inflammatory signaling	Early activation, morphological remodeling, increased responsiveness to glutamatergic and inflammatory signals, onset of proliferation and migration toward the lesion area	Injured neurons and axons, microglia/macrophages, endothelial cells, astrocytes	May contribute to early injury sensing, local tissue stabilization, and initiation of inflammatory and extracellular matrix remodeling responses	[51,53]
Subacute phase	Several days to weeks	Expansion and organization of secondary injury, glial and fibrotic scar formation, inflammatory cell recruitment, demyelination and remyelination attempts, extracellular matrix remodeling	Accumulation in the perilesional region, proliferation, increased NG2/CSPG4 expression, oligodendrocyte lineage progression, context-dependent differentiation into astrocytic phenotypes reported in some models	Reactive astrocytes, microglia/macrophages, spared axons, endothelial/perivascular cells, extracellular matrix components	Dual role: may support remyelination and lesion containment, but may also contribute to CSPG-rich inhibitory matrix formation and restriction of axonal growth	[40,53,54]
Chronic phase	Weeks to months	Cystic cavity maturation, persistent inflammation, chronic demyelination, incomplete remyelination, axonal dieback, long-term extracellular matrix remodeling	Persistence of reactive or incompletely differentiated NG2 glia, region-dependent remodeling, residual or sustained NG2/CSPG4-associated signaling, altered interactions with dystrophic axons and scar components	Dystrophic axons, astrocytic scar border, microglia/macrophages, vascular niche, chronic extracellular matrix	May preserve remyelinating potential, but may also participate in maintenance of inhibitory matrix properties, axonal trapping, and long-term regeneration failure	[4,40,53,54]

**Table 2:** Functional effects of NG2 glia in spinal cord injury (SCI). The table summarizes experimentally supported and proposed functional roles of NG2 glia in SCI based on available evidence. Some mechanisms are derived from related CNS contexts and may not yet be fully validated in the adult spinal cord.

Functional Orientation	Biological Effect	Interacting Cell Types	Cellular and Molecular Mechanisms	Primary Evidence Context	Source
Reparative	Axonal remyelination	Neurons, oligodendrocytes	Oligodendrogenesis, axonal remyelination	SCI model and broader CNS demyelination/remyelination literature	[53,61]
Context-dependent/dual	Glial scar formation	Astrocytes, microglia	NG2 glial proliferation, CSPGs secretion, cooperation with reactive astrocytes and microglia	SCI model	[18,53]
Context-dependent/dual	Differentiation into astrocytes	Astrocytes	Up to 25% of NG2 glial progeny differentiate into GFAP^+^ astrocytes in SCI; this process is context-dependent and has been associated with BMP and Wnt/Shh signaling pathways	SCI lineage-tracing model	[17,19]
Inhibitory	Inhibition of axonal growth	Neurons	NG2/CSPG4 interacts with receptors such as PTPσ and LAR and is associated with inhibition of axonal growth	SCI models and NG2/CSPG4-CSPG axon growth assays	[77]
Inhibitory	Formation of axonal “traps”	Neurons	Non-communicative synapse-like contacts, stabilization of dystrophic endings	SCI model	[4]
Context-dependent	Regulation of proliferation and differentiation	NG2 glia (self-regulation)	Wnt/β-catenin signaling pathway; β-catenin deletion has been associated with reduced NG2 glial proliferation	SCI model, OPC biology studies	[69]
Context-dependent/dual	Immunomodulation	Microglia, macrophages	Sensitivity to inflammatory mediators (e.g., TNF-α, MMP-9); microglia may influence NG2 glial proliferation	SCI model and in vitro microglia/macrophage-OPC co-culture studies; some mechanisms inferred from related CNS inflammation models	[114]
Reparative/context-dependent	Maintenance of vascular and BSCB integrity	Endothelial cells	TGF-β-dependent regulation of tight junction protein expression	Mainly CNS vascular/BBB models; direct validation in SCI/BSCB remains limited	[98]

## Data Availability

No data was used for the research described in the article.

## Abbreviations

The following abbreviations are used in this manuscript:

SCI	Spinal cord injury
OPCs	Oligodendrocyte progenitor/precursor cells
CSPGs	Chondroitin sulfate proteoglycans
NG2/CSPG4	Neuron-glial antigen 2/chondroitin sulphate proteoglycan 4
ECM	Extracellular matrix
CNS	Central nervous system
MMPs	Matrix metalloproteinases
HSPGs	Heparan sulfate proteoglycans
